# Preventing mental health problems in children: the *Families in Mind* population-based cluster randomised controlled trial

**DOI:** 10.1186/1471-2458-12-420

**Published:** 2012-06-08

**Authors:** Harriet Hiscock, Jordana K Bayer, Kate Lycett, Obioha C Ukoumunne, Daniel Shaw, Lisa Gold, Bibi Gerner, Amy Loughman, Melissa Wake

**Affiliations:** 1 Centre for Community Child Health, Royal Children’s Hospital, Parkville, Australia; 2 Murdoch Childrens Research Institute, Parkville, Australia; 3 Department of Paediatrics, University of Melbourne, Parkville, Australia; 4 Psychological Science, LaTrobe University, Melbourne, Australia; 5 PenCLAHRC, Peninsula College of Medicine and Dentistry, University of Exeter, Exeter, Devon, UK; 6 Department of Psychology, University of Pittsburgh, Pittsburgh, PA, USA; 7 Deakin Health Economics, Deakin University, Melbourne, Australia

## Abstract

**Background:**

Externalising and internalising problems affect one in seven school-aged children and are the single strongest predictor of mental health problems into early adolescence. As the burden of mental health problems persists globally, childhood prevention of mental health problems is paramount. Prevention can be offered to all children (universal) or to children at risk of developing mental health problems (targeted). The relative effectiveness and costs of a targeted only versus combined universal and targeted approach are unknown. This study aims to determine the effectiveness, costs and uptake of two approaches to early childhood prevention of mental health problems ie: a Combined universal-targeted approach, versus a Targeted only approach, in comparison to current primary care services (Usual care).

**Methods/design:**

Three armed, population-level cluster randomised trial (2010–2014) within the universal, well child Maternal Child Health system, attended by more than 80% of families in Victoria, Australia at infant age eight months.

Participants were families of eight month old children from nine participating local government areas. Randomised to one of three groups: Combined, Targeted or Usual care.

The interventions comprises (a) the Combined universal and targeted program where all families are offered the universal *Toddlers Without Tears* group parenting program followed by the targeted *Family Check-Up* one-on-one program or (b) the Targeted *Family Check-Up* program. The *Family Check-Up* program is only offered to children at risk of behavioural problems.

Participants will be analysed according to the trial arm to which they were randomised, using logistic and linear regression models to compare primary and secondary outcomes. An economic evaluation (cost consequences analysis) will compare incremental costs to all incremental outcomes from a societal perspective.

**Discussion:**

This trial will inform public health policy by making recommendations about the effectiveness and cost-effectiveness of these early prevention programs. If effective prevention programs can be implemented at the population level, the growing burden of mental health problems could be curbed.

**Trial registration:**

ISRCTN61137690

## Background

Mental health problems account for a substantial burden of disease globally, with the World Health Organisation predicting that by 2030, mental health problems will be the highest ranking disease in terms of burden in affluent countries [1]. Similar to Australian adults (where up to one in five have a mental health problem) [2], one in seven Australian children (aged 4–17) has a mental health problem, yet in a national survey only a quarter of children accessed treatment [3]. The relative lack of child and adolescent mental health services poses a pressing problem as prevalence persists [1]. To curb and manage this problem, effective prevention is essential.

During childhood, mental health problems most commonly manifest as externalising (behavioural) and internalising (emotional) problems [4-8]. Externalising problems include conduct disorders, oppositional defiance and aggression, while internalising problems include anxiety, social withdrawal and depression. These problems bear considerable ongoing costs for individuals, families and society [9] including difficulties with peer interaction, learning, family stress and the need for clinical services [10]. Associated problems can also be enduring; mental health problems in early childhood are the single strongest longitudinal predictor of mental health throughout childhood and into early adolescence [4]. If left untreated, up to 50% of these problems can persist throughout childhood and then adolescence [6], resulting in an increased risk of school dropout, substance abuse, family violence, unemployment, involvement with criminal justice services, and suicide [9,11].

Externalising and internalising problems share early risk factors, many of which are identifiable in the pre-school years [7,12]. Risk factors include family stressors such as parental mental health problems, single parenthood, substance abuse, relationship conflict, social isolation, low income and maternal perception of difficult child temperament [7,12]. The single strongest modifiable risk factor, however, is negative parenting practices [13,14]. Negative parenting practices characterised by harsh discipline and low warmth are predictive of externalising problems, and over-involved protective parenting and low warmth are predictive of internalising problems [12,15]. Thus, a focus on parenting practices is an essential component of prevention programs for mental health problems in childhood.

### Universal and targeted approaches to prevention of child mental health problems

Two broad types of prevention programs exist: universal (i.e. provided to all) and selective or targeted (i.e. provided to ‘at risk’ populations), but in reality the boundaries between these two approaches are often blurred [16]. Our systematic review of randomised controlled trials of early intervention and prevention programs for child mental health revealed a number of targeted programs that reduced externalising problems in randomised controlled trials [17]. These include the Olds Home Visiting Program (an intensive program promoting maternal health and a good parent-infant relationship in the first two years of life) that has shown lasting reductions in anti-social adolescent behaviour [18], yet lacks demonstrated comparable efficacy when translated to the Australian population [19,20]. Similarly, *The Incredible Years* program has shown reductions in externalising behaviours [17] and more recently has shown promise for reducing co-occurring internalising problems in children aged 4–7 years with existing oppositional defiant disorder [21]. However, both of these programs are limited by their resource intensity with a minimum of 40 contact hours per family [17,21]. The most promising targeted program, in terms its brevity and effectiveness, is the *Family Check-Up* program (detailed below in Methods). The *Family Check-Up* provides a relatively small number of one-on-one sessions (an average of 3.3 sessions per family) to ‘at-risk’ families i.e. those experiencing child behaviour problems and/or economic and family hardship. It has proven effective in preventing both externalising and internalising problems [22]. However, it has only been trialed in disadvantaged American families. Yet child mental health problems occur across all socioeconomic groups and numerically the bulk of problems occurs in middle and high socioeconomic groups in many countries, because these groups comprise the bulk of society [17,23]. There is a need, therefore, to test the efficacy of the *Family Check-Up* in countries other than the US, across a range of socioeconomic groups.

Although often effective, targeted programs can be stigmatising for families and lead to poor uptake rates, as low as 20% in some studies [23]. An alternative approach to improve the reach of targeted programs may be to offer a universal prevention program first. Universal programs include *Triple P* (i.e. Positive Parenting Program) which has been trialed in both Australia [24] and Germany [25]. In these two trials, *Triple P* involved four weekly 2-hour parenting groups plus optional 15-minute phone contacts for parents of children aged 3–6 years. Improvements in parenting, child behaviour and family stress have been reported [24,25]. Neither trial however, delivered *Triple P* in a truly universal manner [24,25]. The recruitment rate for the German trial was 31% of the population and a high proportion of these children (32%) had pre-existing behaviour problems [25]. The Australian trial was restricted to families from low socioeconomic areas, was not randomised and nearly half of the children had pre-existing behaviour problems. Population recruitment rates need to be higher in universal prevention trials for generalisability, interpretation of effect sizes and understanding the logistics of program dissemination [24].

The *Toddlers Without Tears* program is one of the few truly universal mental health prevention programs [15,26,27]. Developed in Australia to address negative parenting styles that can contribute to child mental health problems, it consists of a nurse delivered one-on-one session at child aged 8 months followed by two parent group sessions delivered at child age 12 and 15 months by maternal and child health nurses and a co-facilitator with expertise in parenting. In a large (N = 733) randomised controlled trial with high recruitment (69% of the population), the program led to some modest improvements in parenting practices but did not prevent behavioural and emotional problems in preschoolers [26,27]. This suggests that this program alone is insufficient to prevent child mental health problems. Whether this program combined with a targeted program could lead to greater population reach, uptake and effectiveness remains to be determined. Given that no trial to date has evaluated the effects of a combined universal-targeted approach versus a targeted approach alone, this trial’s findings are likely to be of international significance.

The *Families in Mind* trial therefore aims to compare the effectiveness, cost-effectiveness and population reach and uptake of a targeted approach alone (the *Family Check-Up* program) with a combined universal (the *Toddlers Without Tears* group parenting sessions) and targeted approach in the Australian population. Both programs will be delivered through existing child health workforces in the state of Victoria, and will be compared to the provision of usual care alone (‘control’ group).

We hypothesise that families offered this targeted program, either alone or in combination with this universal program, will have better outcomes than families who are not offered these programs. Outcomes include mean scores at child age three, four and five years for:

a) child externalising and internalising behaviour problem s (primary outcome)

b) harsh discipline and nuturing parenting practices (primary outcome), and

c) parental mental health (secondary outcome)

Additionally, we hypothesise that uptake of the targeted program by ‘at risk’ families will be greater with the combined approach where the universal parenting program precedes the targeted program (to reduce stigma), than with the targeted program alone.

## Methods/design

**Figure 1:**
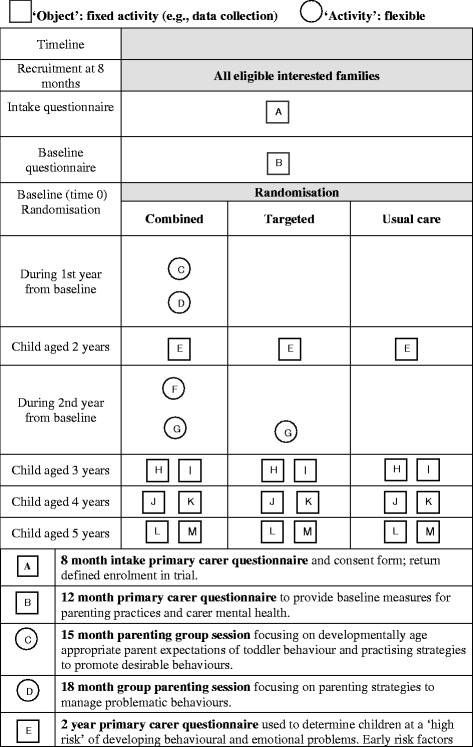
Graphical depiction of components of the trial.

Figure 1 summarises the components of the trial and their timing. It graphs each stage of all three arms of the trial, in the manner suggested by Perera et al. [28]. The trial is registered with an international trial registry (ISRCTN61137690) and will be reported in accordance with the CONSORT statement [29].

### Design

Three-arm cluster randomised controlled trial comprising Arm A (combined approach of *Toddlers Without Tears* program followed by the *Family Check-Up* program); Arm B (*Family Check-Up* program only); and Arm C (Usual care) (see Figure 1). The trial runs from 2010 to 2014.

### Funding and ethics approval

This trial is funded by a Partnership Grant from the National Health and Medical Research Council of Australia (project grant number: 546525) and has been granted ethics approval by the Royal Children’s Hospital (#29144) and Deakin University (#2010-156) Human Research Ethics Committees.

### Recruitment process

To develop the sampling frame, the 31 local government areas (LGAs) comprising greater Melbourne (population 3,592,591 in 2006), Australia, were first ranked according to the mean score of all inhabitants on the census-based Socio-Economic Indexes for Areas (SEIFA) index of relative disadvantage [30], and then divided into tertiles. A convenience sample of three local government areas from each of the low, middle and high tertiles was then approached to take part in the trial.

Maternal and child health (MCH) nurses in these areas then consecutively invited families attending their routine 8-month well-child visit over a five-month period (Aug10-Dec10) to learn more about the study. The MCH service is a universal primary health service available free to families in every local government area in Victoria. It provides ten ‘Key Ages and Stages’ visits with an MCH nurse from birth to school entry, with 84% attendance at the 8-month MCH visit [31]. Nurses passed interested families’ contact details on to the research team, who then contacted the family by phone. Families who did not attend their scheduled 8-month visit were sent a letter inviting them to contact the research team for more information about the study.

During the recruitment call, the research team provided background information and a description of the programs being trialed. Families interested in participating were sent an enrolment pack containing the participant information statement, consent form and the 8-month intake survey. Participants enrolled when a signed consent form and an intake questionnaire were returned by post.

### Inclusion criteria

As this is a population-level effectiveness trial for programs delivered in a community setting, inclusion criteria were as broad as possible: all 8-month old babies who attended or planned to attend their MCH service in the participating local government areas between August 2010 and December 2010.

### Exclusion criteria

Parents with insufficient spoken English to participate in parenting programs were excluded. This was determined by the referring nurse or the research team at the recruitment phone call. Infants with major medical diagnoses were also excluded.

### Allocation

MCH centres (n = 133) were grouped into clusters (n = 85) before randomisation to avoid cross-contamination of new intervention program skills, which could have occurred when a nurse worked across multiple centres - had these centres not been allocated to the same trial arm. Randomisation of clusters was stratified by LGA. Clusters were rank-ordered within LGA according to the number of participants recruited and block randomisation was used with fixed block sizes of three to minimise the imbalance in the number of participants in each of the three trial arms. Randomisation was performed after the recruitment phase by a statistician independent of the recruitment process. Families and nurses were notified of their group allocation in writing.

### Interventions

#### Content

The universal program is a revised version of the *Toddlers Without Tears* program that incorporates feedback from the previous trial [26,27]. Based on parent feedback, the group sessions are extended (to include strategies around prevention of internalising behaviours) and delivered around child age 15, 18 and 24 months (rather than the original 12 and 15 months), as this better matches the toddler period when most children become mobile and more challenging in their behaviours. The information covered in these three group sessions provides evidence-based guidance on developmental expectations of toddler behaviour, strategies to encourage desirable behaviours (e.g. praise and rewards) and strategies to manage problematic behaviours (e.g. ignoring, logical consequences, distraction, quiet time and anxiety desensitization) [27].

The *Family Check-Up*[22] is a one-on-one family support program consisting of in-home sessions with a ‘parent consultant’ – a trained psychologist. The *Family Check-Up* aims to address problems in the family environment known to impact on children’s behavioural and emotional development [22]. The *Family Check-Up* will be offered to families in the Combined and Targeted groups with toddlers identified as ‘at risk’. Children will be deemed at risk if they score over one standard deviation above the normative mean at child age two years for a) externalising problem behaviour scores on the Child Behavior Checklist (CBCL) [32] and/or b) the Inhibitory Control subscale on the Children’s Behavior Questionnaire and/or c) the Harsh Discipline subscale on the Parent Behavior Checklist [14]. Families that accept will then be visited by a parent consultant specifically trained in the program, who will follow Shaw, Dishion and colleagues’ procedure for profiling family strengths and difficulties by motivational interviewing assessment/intervention. The *Family Check-Up* consists of an initial ‘Get to Know You’ session with the child’s primary caregiver/s which includes an interview and observational assessment task. At this session, the parent consultant will ask families for written consent to (1) use their questionnaire data for family profiling, and (2) video record some parent–child interactions. During the second ‘Feedback’ session, the parent consultant presents detailed integrated assessment results of child and family strengths and difficulties on a clear visual Child and Family Profile [33]. If the toddler is ‘at risk’ of developing behaviour and/or emotional problems on the Child and Family Profile, families will be offered up to four further ‘Intervention’ sessions focusing primarily on parenting skills and related family stressors, with referrals to appropriate services in the community for additional support as required (e.g. housing support, drug and alcohol advice).

### Process: combined arm

The first program of the Combined arm will be *Toddlers Without Tears,* the series of three parenting group sessions running for approximately 2 hours each, offered to all families randomised to this arm. An MCH nurse and psychologist with experience in facilitating parenting groups will deliver each session to groups of 4–12 parents. The sessions involve role plays with practice activities, multi-choice scenario discussions, parent education activities utilising parent hand outs, and summarising ideas to practice at home directly with toddlers. All sessions are supported by *Toddler without Tears* parent handouts. Parents who do not attend a session are mailed the relevant parent handouts and offered a 30 minute telephone consultation with a psychologist to discuss the key messages provided in the group session and handouts.

Families in this Combined arm group that are identified with a child ‘at risk’ at age two years will be offered the *Family Check-Up.* We estimate that ≈ 10% of families will be eligible for the *Family Check-Up*; these families will receive a phone call from the research team inviting them to participate in the *Family Check-Up* program. Families will be free to accept or decline this offer.

### Process: targeted arm

Families in the Targeted arm will be offered the *Family Check-Up* program if their child is found to be ‘at risk’ at 2 years of age, as per the process detailed above. Eligibility criteria and intervention will proceed in the same way as for the Combined arm. It is anticipated that *Family Check-Up* uptake in this Targeted arm will be lower than in the Combined arm, as all families in the Combined arm will have been offered some help through the *Toddlers Without Tears* program and may find the recruitment approach into the *Family Check-Up* a more natural extension of assistance.

### Process: usual care arm

Families in the Usual care arm will receive only usual care from their MCH nurse and other health and social services in their community. This does not include the *Toddlers Without Tears* or *Family Check-Up* program.

### Outcome measures

Outcomes will be multi-source, collected from both primary and secondary carers. All families complete an intake questionnaire at child age 8 months and a baseline questionnaire at child age 12 months, and will be asked to complete one postal questionnaire annually around the child’s birthday until child age five years. The 8-month questionnaire measures child demographic characteristics (age, gender), family characteristics (marital status, parent age, education level, country of birth, main language spoken at home) as well as potential confounders including: (1) psychosocial risk factors measured by the Family Psychosocial Screening Instrument (12 items, public health screen for domestic violence, parent substance abuse, social isolation) [34]; (2) conflicts over child-rearing measured by the Parenting Problem Checklist (17 items) [35]; (3) relationship dissatisfaction measured by the Partner Relationship Scale (7 items) [36]; and infant temperament measured by the Maternal Child Difficulty Rating (1 item) [37]. Baseline questionnaire measures are described in Table 1.

**Table 1:** Secondary outcome measures and time-points

**Construct**	**Measure**	**Administration time points (child age in yrs)**					**Rationale for use**
		Baseline*	**2y**	**3y**	**4y**	**5y**	
**Parent questionnaire measures:** Primary carer ■ Secondary carer ▴							
Child major health or developmental diagnoses	Parents’ Evaluation of Developmental Status (PEDs) [38]	■					Children with major diagnoses are excluded.
Child quality of life	PedsQL 4.0 Generic Core Scale [39]			■	■	■	Child health-related quality of life.
Parenting practices	Over involved/protective parenting scale [12]	■	■	■ ▴	■ ▴	■▴	Assessment of parenting practices.
Parent mental health	Depression Anxiety Stress Scales (DASS21) [40]	■	■	■▴	■ ▴	■▴	Impact on parental mental health.
Parent quality of life	Assessment of Quality of Life 6D [41]	■	■	■	■	■	Independent living, mental health, coping, relationships, pain and senses.
Costs	Child and adult health service use		■	■▴	■ ▴	■▴	Intervention process evaluation (policy/decision makers considering translation/dissemination).
Feedback: *Family Check-Up*	Parents acceptability and usefulness rating of the intervention			■			Intervention process evaluation (translation/dissemination uptake by families).
Feedback: *Toddlers Without Tears*	Parents acceptability and usefulness rating of the intervention		■	■			Intervention process evaluation (translation/dissemination uptake by families).
**Health professional measures: MCH nurses▴**							
Construct	Measure	Administration time points (child aged yrs)					Additional information
		15 & 18 months	2	3	4	5	
Delivery/Fidelity *Toddlers Without Tears* Program	Group sessions content checklists	**▴**	**▴**				Integrity of intervention delivery. Adapted for this study.

* Baseline includes the 8 and 12 month questionnaires.

We have two primary outcome measures: the Child Behavior Checklist (CBCL/1.5-5 years) – a widely used and validated 99-item measure of externalising and internalising behaviours [32] and the nurturing and harsh discipline subscales of the Parenting Behavior Checklist [14]. All primary outcomes will be measured at 4 time points. All secondary measures and process evaluation measures are reported in Table 1.

### Prognostic factors

We will adjust analyses for potential prognostic factors measured in the 8-month questionnaire.

### Economic evaluation

Costs of delivering the *Toddlers Without Tears* and *Family Check-Up* programs (e.g. materials and training) will be measured largely through research team and MCH records. Costs of families’ use of health and other services outside of the study (e.g. other psychologists, psychiatrists, media resources) will be measured by parental report. Measured resource use will be valued using existing unit cost estimates (e.g. Medicare fee schedule rates). Economic evaluation will be presented first as a cost-consequences analysis [42], which allows policy makers to compare the incremental costs with all outcomes of interest – i.e. child behaviour, parenting, caregiver mental health and impact on health-related quality of life. Economic evaluation will then present a cost-utility analysis, comparing incremental costs to incremental parental quality of life (as measured by AQoL-6D) [41].

### Power calculation and study population

The sample size is based on detecting a reduction of 0.25 of a standard deviation (SD) in the mean scores for externalising behaviour problems on the CBCL at age 5 years with 80% power and 2-sided significance level of 0.05. Ignoring clustering effects, 252 children would be required in each of the three trial arms (756 in total). As nine LGAs are included in the study, we anticipated from our previous trial [26] that 60 MCH clusters would be recruited with 20 of these allocated to each trial arm. For an individually randomised trial 12.6 (756/60) children would need to be recruited from each MCH centre. In order to allow for correlation between the responses of children from the same cluster [43], we need to inflate this figure using a formula provided by Campbell [44] that is appropriate when the number of clusters is fixed and known in advance, but the number of participants required per cluster needs to be calculated. Using this formula and assuming an intra-cluster (intra-MCH centre) correlation coefficient of 0.03 (estimated from our previous trial) for the CBCL externalising behaviour problems outcome, 393 subjects are required in each trial arm [27]. Allowing for 20% attrition by age five, we need to recruit 492 children in each trial arm (1476 in total).

### Analyses

We will compare mean SIEFA scores and child gender for those eligible families who chose to participate vs those who chose not to participate. We will also describe the reasons they chose not to take part including ‘too busy’, ‘not interested’, and ‘moving house’. Demographic and baseline characteristics at the MCH cluster and family levels will be summarised using means and standard deviations (or medians and inter-quartile ranges) for quantitative characteristics and percentages for categorical characteristics. Analyses will use the ‘intention to treat’ principle, with families that provide outcome data analysed according to the trial arm to which their MCH centre was randomised. All analyses will allow for correlation between outcome scores of participating families from the same MCH cluster. Primary analyses will compare the mean CBCL externalising and internalising scores reported by the primary caregiver at ages 3, 4 and 5 years across the three trial arms.

Secondary analyses will compare the CBCL scores reported by the secondary caregiver and, for both caregivers, compare mean parenting subscale scores (nurturing, harsh discipline, inappropriate expectations, over-involved/protective), mean parental depression, anxiety and stress scores, and mean parent quality of life scores across the trial arms. Mean health care costs reported by the primary caregiver will also be compared between trial arms. Tests of interaction will be used to investigate whether the intervention effects differ between outcomes reported by the primary and secondary caregivers. If there is evidence at the 5% level of significance of differential effects then separate effect sizes will be reported for each caregiver, otherwise a single overall effect size will be reported.

Analyses of quantitative outcomes (unadjusted and adjusted for potential prognostic factors measured at baseline) will be implemented using random effects linear regression fitted using restricted maximum likelihood estimation [45]. Intra-cluster (intra-MCH centre) correlation coefficients will be reported for the study outcomes to aid the planning of future cluster randomised trials in this area. The take up of the *Family Check Up* program (binary outcome) amongst ‘at risk’ families will be compared between the Combined and Targeted arms by marginal logistic regression models using Generalised Estimating Equations with information sandwich estimates of standard error and assuming an exchangeable correlation structure [46].

## Discussion

Early intervention may prove crucial to managing the burden of mental health problems in children and adults. Few studies have examined the effectiveness and feasibility of truly universal prevention programs alone, while no studies have conducted a randomised control trial to evaluate a combined universal program followed by a targeted program. We will assess whether the effectiveness, uptake and reach of a targeted family support program can be enhanced by the prior delivery of a brief universal parenting group intervention. This will be measured by participation rates and improvements in child behaviour, parenting practices and parent mental health measures across the three groups.

This randomised controlled cluster trial will inform public health policy and make recommendations about the effectiveness and cost-effectiveness of these early prevention programs. If effective prevention programs can be implemented at the population level, then the burden of mental health problems could be curbed.

## Abbreviations

LGA, Local government area; MCH, Maternal and child health; CBCL, Child behavior check list.

## Competing interests

All authors declare that HH, JB, KL, OU, DS, LG, BG, AL, MW, their spouses, partners or children have no financial and non-financial relationships or interests that may be relevant to the submitted work. The authors declare they have no competing interests.

## Authors’ contributions

HH, MW, JB and OU conceived the study. KL, BG and AL drafted the current manuscript. HH, JB, OU, DS, LG, MW participated in the design of the study. All authors have contributed to and edited the current manuscript. All authors have read and approved the final manuscript.

## Pre-publication history

The pre-publication history for this paper can be accessed here:

http://www.biomedcentral.com/1471-2458/12/420/prepub
